# Advances in pharmacological research on myocardial remodeling agents: A decade in review

**DOI:** 10.1097/MD.0000000000042757

**Published:** 2025-06-06

**Authors:** Jimin Liu, Xiaqing Tian, Meng Zhang, Jiaxuan Li, Yongjun Chen, Taiyi Wang

**Affiliations:** a Innovation Research Institute of Chinese Medicine, Shandong University of Traditional Chinese Medicine, Jinan, People’s Republic of China; b College of Animal Science and Veterinary Medicine, Shandong Agricultural University, Tai’an, People’s Republic of China; c Institute of Acupuncture and Moxibustion, Shandong University of Traditional Chinese Medicine, Jinan, People’s Republic of China; d Shandong Key Laboratory of Innovation and Application Research in Basic Theory of Traditional Chinese Medicine, Shandong University of Traditional Chinese Medicine, Jinan, People’s Republic of China; e Key Laboratory of Traditional Chinese Medicine Classical Theory, Ministry of Education, Shandong University of Traditional Chinese Medicine, Jinan, People’s Republic of China.

**Keywords:** cardiac remodeling, multicomponent agents, multitarget therapy, natural products

## Abstract

Myocardial remodeling, a complex adaptive response to pathological stimuli, plays a pivotal role in the progression of various cardiovascular diseases, including arrhythmias and heart failure. Significant progress has been made in understanding the molecular mechanisms of myocardial remodeling and exploring the efficacy of single and multicomponent agents. Myocardial remodeling entails a complex signaling network that incorporates certain identified critical “bridge nodes,” which are also significant drug targets in classical pharmacological approaches. Nonetheless, some multicomponent drugs that have undergone clinical trials, such as Qili Qiangxin capsules, may suggest the presence of a new and feasible drug development path. The potential of multicomponent agents lies in their ability to achieve a synergistic effect through the coordinated regulation of multiple up- and downstream molecules in signaling pathways involved in myocardial remodeling. However, the development of multicomponent agents presents several challenges, such as identifying active compounds, defining their mechanisms of action, and determining the optimal proportions of each component. Delving deeper into the synergistic, multitarget effects of multicomponent agents in the realm of future research holds the promise to chart a new course toward the development of more effective and safer therapeutic strategies for managing myocardial remodeling and its associated cardiovascular diseases.

## 1. Introduction

Myocardial remodeling represents a complex adaptive process initiated in response to pathological hemodynamic stress, manifesting as coordinated alterations across multiple biological hierarchies. This process involves the reorganization of cardiomyocytes, the extracellular matrix, and the coronary vasculature. Notable structural changes include ventricular wall thickening, cardiomyocyte hypertrophy, and interstitial fibrosis. Functionally, remodeling is characterized by modifications in cardiac output and contractility. At the molecular level, it involves the dysregulation of signaling pathways, leading to apoptosis and necrosis of cardiomyocytes.^[1]^ While cardiac remodeling is a protective response to stress or injury, prolonged or excessive remodeling can lead to severe cardiac damage, dysfunction, malignant arrhythmias, and heart failure.^[2]^ This creates a pathological feedback loop where diseases initiate and exacerbate remodeling. Understanding these mechanisms is crucial for effective prevention and treatment of cardiovascular diseases.

Cardiac remodeling significantly affects the development and progression of diseases such as arrhythmias and heart failure. Arrhythmias, especially atrial fibrillation (AF), are associated with myocardial remodeling, with the rapid heart rate associated with long-term AF resulting in hypertrophy of the ventricles and increased endomyocardial fibrosis.^[3]^ Cardiac remodeling can lead to cardiomyopathy and myocardial fibrosis, which, in turn, can cause arrhythmias.^[4]^ Cardiomyopathy is a heart disease characterized by abnormal structure or function of the myocardium, associated with hypertrophy or dilation of the ventricles in the heart. In all types of cardiomyopathy, the annual incidence of sudden death in patients ranges from 0.15% to 0.7%.^[5]^ Cardiac remodeling is also a precursor stage and integral component of heart failure, characterized by a decrease in the heart’s ability to pump blood or fill with blood, both of which are caused by structural or functional abnormalities of the heart.^[6]^ According to current estimates, in 2017, an estimated 64.3 million people worldwide were living with heart failure.^[7]^ In a study conducted in the United States on elderly individuals aged 65 and older, it was demonstrated that the mortality rate due to heart failure within 1 year reached a staggering 33%.^[8]^ Additionally, hypertensive heart disease, caused by uncontrolled high blood pressure, primarily affects the left side of the heart and the coronary arteries.^[9]^ Elevated blood pressure induces abnormal mechanical stress on the myocardium, causing ventricular dysfunction, cardiomyocyte apoptosis, and myocardial fibrosis.^[10]^ Myocardial infarction (MI), resulting from coronary artery obstruction, triggers cardiac remodeling. This remodeling occurs in 2 stages: early (within 24–72 hours) and late (over several weeks), involving cardiomyocyte hypertrophy, apoptosis, and fibrosis.^[11]^ Besides the aforementioned, some medications can induce heart disease. For example, doxorubicin, used in cancer treatment, causes cardiotoxicity, leading to cardiomyopathy and heart failure.^[12]^

The molecular mechanisms of myocardial remodeling include cardiomyocyte death, hypertrophy, extracellular matrix alterations, fibrosis, and mitochondrial dysfunction.^[13]^ These changes are compensatory mechanisms for the heart under external stress and volume overload. Overactivation of the renin-angiotensin system (RAS) is a key factor, with angiotensin II inducing structural and functional heart impairments. Experimental studies have found that angiotensin II and its downstream effector, aldosterone, significantly increase the expression of Toll/interleukin (IL)-1 receptor domain-containing adapter-inducing IFN-β, accompanied by abnormal cardiac structure and impaired cardiac function. The mechanism involves Toll/IL-1 receptor domain-containing adapter-inducing IFN-β–mediated nuclear factor (NF)-κB transcriptional regulation and downstream activation of the epidermal growth factor receptor (EGFR) signaling pathway through EGFR ligands.^[14]^ Cardiomyocyte hypertrophy involves an increase in cell volume, diameter, and sarcomeres. Fibroblast growth factor 18 and its downstream factor tyrosine protein kinase (FYN) play a significant role in pressure overload-induced hypertrophy. Fibroblast growth factor 18 promotes FYN activity and expression while regulating nicotinamide adenine dinucleotide phosphate (reduced form) oxidase 4, reducing reactive oxygen species and alleviating hypertrophy.^[15]^ During isoproterenol- and aldosterone-induced hypertrophy, miR-23 expression is upregulated, modulated by the calcium-neurogenic hypertrophic pathway, which regulates cardiomyocyte hypertrophy by downregulating muscle-specific ring-finger protein 1 expression.^[16]^ Understanding myocardial remodeling mechanisms and their link to cardiovascular diseases is essential.

This article reviews recent advancements in pharmacological treatments for myocardial remodeling, evaluating current therapies’ efficacy and limitations. Specifically, we emphasize the concept of multitargeted therapy for myocardial remodeling and identify critical signaling hubs within the mechanism and target networks that are crucial for therapeutic intervention. These evolutionarily conserved hubs, such as transforming growth factor(TGF)-β /Smad3, NF-κB, and microRNAs (miRNAs), serve as molecular gatekeepers coordinating fibrotic, inflammatory, and metabolic reprogramming through their privileged positions in signaling crosstalk. By refining our focus on these strategic elements that integrate multiple pathological pathways, the review aims to enhance therapeutic coherence and contribute to the advancement of cardiovascular disease treatment and prevention strategies. It also explores new drug development directions targeting these hierarchical control points, along with future research opportunities in deciphering their dynamic interactions, aiming to improve cardiovascular disease management.

## 2. Drugs for improving myocardial remodeling

### 2.1. Mono-component agents for improving myocardial remodeling

In cardiology, research into mono-component drug therapy for myocardial remodeling is progressing, with increasing interest in the diverse and multitargeted effects of mono-component agents (most of which are natural products). This section examines the mechanisms of action of several key monomeric compounds used in treating myocardial remodeling (as shown in Table 1).

**Table 1:** Mono-component agents and related pharmacological studies on improving myocardial remodeling.

Compound	Source	Animal	Model	Chemical Structure	Mechanism	References
Astragaloside IV derivative HHQ16	Radix Astragali	Mice	LADL-induced HF	C_30_H_48_F_2_O_4_	lnc4012/9456↓, f G3BP2↓, IkB α↓, NF-kB p65.	^[17]^
Astragaloside IV	Radix Astragali	Mice	Dox-induced HF	C_41_H_68_O_14_	Nrf2↑, HO-1↓.	^[18]^
Astragaloside IV	Radix Astragali	Mice	LADL-induced MI	C_41_H_68_O_14_	ROS↓, Caspase-1↓, GSDMD↓.	^[19]^
Astragaloside IV	Radix Astragali	Mice	ISO-induced CF	C_41_H_68_O_14_	Akkermansia↑, Defluviitaleaceae_UCG-011↑, Rikenella↑.	^[20]^
Ginsenoside Rg1	Panax ginseng	Mice	LADL-induced MI	C_42_H_72_O_14_	SIRT1↑, PINK1↑.	^[21]^
Ginsenoside Rg1	Panax ginseng	Mice	DIC	C_42_H_72_O_14_	TIF1A↓, p-P70S6K↓, JNK1↓, Beclin-1↓, GRP78↑.	^[22]^
Ginsenoside Rg2	Panax ginseng	Rats	ISO-induced CF	C_42_H_72_O_13_	TGF-b1↓, Smad↓.	^[23]^
Ginsenoside Rg3	Panax ginseng	Mice	TAC-induced CH	C_42_H_72_O_13_	The 2-hydroxyisobutylation levels of DLD↓, P300↓.	^[24]^
Ginsenoside Rb1	Panax ginseng	Mice	LADL-induced MI	C_54_H_92_O_23_	Mitophagy↑.	^[25]^
Ginsenoside Rb1	Panax ginseng	Mice	LADL-induced MI	C_54_H_92_O_23_	Mitochondrial complex I↓, NADH dehydrogenase ↓.	^[26]^
Ginsenoside Rb3	Panax ginseng	Mice	HF postmyocardial infarction model	C_53_H_90_O_22_	NF-κB p65↓, SERCA2a↑.	^[27]^
Ginsenoside Re	Panax ginseng	Mice	LADL-induced AMI	C_42_H_72_O_13_	Myd88↓, NF-kB p65↓.	^[28]^
Notoginsenoside R1	Panax notoginseng	Mice	LADL-induced MI	C_47_H_80_O_18_	TAK1↓, JNK ↓, p38↓.	^[29]^
Liquiritin	Glycyrrhiza glabra	Mice	Ang II-induced hypertrophy	C_21_H_22_O_9_	cAMP↑, PKA↑, mTORC1↓, ACC ↓.	^[30]^
Liquiritigenin	Glycyrrhiza glabra	Mice	ISO-induced MI	C_20_H_22_O_9_	ICa-L↓, Damping intracellular Ca^2+^↓.	^[31]^
Liquiritigenin	Glycyrrhiza glabra	Mice	LADL-induced MI	C_20_H_22_O_9_	miR-185-5p↑, CDK6↓.	^[32]^
Glycyrrhizic acid	Glycyrrhiza glabra	Cell line and rats	H9C2 cell-induced by H/R Rats: LADL for 0.5 h followed by 2 h of reperfusion	C_42_H_62_O_16_	ERS-related factors↓.	^[33]^
Glycyrrhizic acid	Glycyrrhiza glabra	Mice	DIC	C_42_H_62_O_16_	Nrf2↑, HO-1↑.	^[34]^
Glycyrrhizic acid	Rats	Rats	LADL for 0.5 h followed by reperfusion	C_42_H_62_O_16_	YAP↓, Hippo↓.	^[35]^
Isoliquiritigenin	Glycyrrhiza glabra	Mice	AB-induced CF	C_21_H_20_O_10_	AMPKα↑.	^[36]^
Isoliquiritigenin	Glycyrrhiza glabra	Mice	LADL-induced AMI	C_21_H_20_O_10_	Nrf2↑, HO-1↑.	^[37]^
Tanshinone I	Salvia miltiorrhiza	Rats	LADL for 0.5 h followed by 2 h of reperfusion	C_20_H_18_O_3_	RIP1↓, RIP3↓, MLKL↓, Akt↑, Nrf2↑.	^[38]^
Tanshinone I	Salvia miltiorrhiza	Mice	DIC	C_20_H_18_O_3_	Nrf2↑.	^[39]^
Tanshinone I	Salvia miltiorrhiza	Mice	ISO-induced MD	C_20_H_18_O_3_	Nrf2↑, MAPK↓, P38 MAPK↓, SAPK/JNK↓, ERK1/2↓.	^[40]^
Tanshinone IIA	Salvia miltiorrhiza	Rats	LADL-induced MI	C_20_H_18_O_4_	Bcl‑2↑, Bak↑.	^[41]^
Tanshinone IIA	Salvia miltiorrhiza	Rats	LADL for 1 h followed by 2 h of reperfusion	C_20_H_18_O_4_	CCR2↓.	^[42]^
Tanshinone IIA	Salvia miltiorrhiza	Mice	DOX-induced	C_20_H_18_O_4_	DAXX↑, MEK↑, ERK1/2 ↑.	^[43]^
Tanshinone IIA	Salvia miltiorrhiza	Mice	TAC-induced CH	C_20_H_18_O_4_	alkB homolog 5↓.	^[44]^
Tanshinone IIA	Salvia miltiorrhiza	Rats	HF-induced by AMI established via LADL	C_20_H_18_O_4_	TLR4↓, NF-κB p65↓.	^[45]^
Cryptotanshinone	Salvia miltiorrhiza	Cell line	A Dox-stimulated H9C2 cell model.	C_19_H_20_O_3_	GSK-3β-ANT↑, mPTP↓.	^[46]^
Cryptotanshinone	Salvia miltiorrhiza	Mice	LADL-induced I/R	C_19_H_20_O_3_	MAPK↓.	^[47]^
Cryptotanshinone	Salvia miltiorrhiza	Mice	AngII-induced CH	C_19_H_20_O_3_	STAT3↓, TGF-β↓, SMAD3↓.	^[48]^
Dihydrotanshinone I	Salvia miltiorrhiza	Mice	DIC	C_21_H_20_O_4_	TFEB↑, p-IKKα/β↓, p-NF-κB↓.	^[49]^
Berberine	Coptis chinensis	Rats	DIC	C_20_H_27_NO_5_	Nrf2↑, HO-1↑, TFAM↑.	^[50]^
Berberine	Coptis chinensis	Rats	TAC-induced CH	C_20_H_27_NO_5_	M-TOR ↓.	^[51]^
Palmatine	Coptis chinensis	Mice	DIC	C_20_H_21_NO_4_	Sirtuin1 ↓.	^[52]^
Palmatine	Coptis chinensis	Mice	ISO-induced CF	C_20_H_21_NO_4_	STAT3↓.	^[53]^
Palmatine	Coptis chinensis	Mice	LADL-induced AMI	C_20_H_21_NO_4_	Inflammatory response↓.	^[54]^
Icariin	Epimedium Genus	Rats	LADL-induced MI	C_33_H_40_O_15_	TGF-β↑, IL-13↑, p-Smad2↑, and p-Smad3↑.	^[55]^
Leonurine	Herba leonuri	Mice	Ang II-induced hypertensive	C_20_H_24_N_2_O	MAPK↓, NF-κB ↓.	^[56]^
Leonurine	Herba leonuri	Mice	Ang II-induced MF	C_20_H_24_N_2_O	p53 ↑, miR-29a-3p ↑.	^[57]^
Leonurine	Herba leonuri	Mice	LADL-induced MI	C_20_H_24_N_2_O	MiR-29a-3p↑.	^[58]^
Luteolin	Melilotus suavcolen	Rats	TAC-induced CH	C_15_H_10_O_6_	PPARγ ↓.	^[59]^
Oxymatrine	Sophora flavescens	HL-1 cell line	HL-1 cells-induced by ISO.	C_21_H_21_NO_9_	TNF-α↓, IL-6↓, TLR4↓, NF-κB↓, MAPK↓.	^[60]^
Aloin	Genus Aloe	Mice	TAC-induced CH	C_21_H_22_O_9_	TGF-β↓, pSmad2/3↓.	^[61]^
Daphnetin	Daphne	Mice	TAC-induced CH	C_9_H_6_O_4_	α-SMA↓, collagen I↓, collagen III↓, TGF-β1↓, Smad2/3↓.	^[62]^

↓ indicates a decrease and ↑indicates an increase.

AB = aortic banding, AMI = acute myocardial ischemia, CF = cardiac fibrosis, CH = cardiac hypertrophy, DIC = doxorubicin-induced cardiomyopathy, DLD = dihydrolipoamide dehydrogenase, Dox = doxorubicin, H/R = hypoxia/reoxygenation, HF = heart failure, HO-1 = heme oxygenase-1, IL = interleukin, ISO = isoprenaline, LADL = left anterior descending coronary artery ligation, MD = myocardial damage, MI = myocardial infarction, mPTP = mitochondrial permeability transition pore, Nrf2 = nuclear factor erythroid 2–related factor 2, PINK1 = PTEN-induced putative kinase 1, PPARγ = peroxisome proliferator-activated receptor γ, SIRT1 = silent information regulator 1, TAC = transverse aortic constriction, TGF-β1 = transforming growth factor beta 1, TLR4 = toll-like receptor 4, YAP = Yes-associated protein.

#### 2.1.1. Nrf2/HO-1 signaling pathway

Glycyrrhetinic acid activates the nuclear factor erythroid 2–related factor 2 (Nrf2)/heme oxygenase-1 (HO-1) signaling pathway, reducing oxidative stress, mitochondrial dysfunction, and apoptosis, thereby preventing doxorubicin-induced cardiotoxicity.^[34]^ Similarly, astragaloside IV regulates the Nrf2/HO-1 signaling pathway in a doxorubicin-induced heart failure rat model.^[18]^ Isoliquiritigenin activates the Nrf2/HO-1 signaling pathway in a mouse model of myocardial ischemia, reducing myocardial oxidative stress and inflammation.^[37]^ Tanshinone I protects against cardiac remodeling in the myocardial ischemia model by activating the Akt/Nrf2 pathway.^[38]^ It also mitigates doxorubicin-induced oxidative stress and apoptosis in cardiomyocytes and protects mitochondria by regulating the Akt/Nrf2 signaling pathway.^[39]^ In a mouse model of isoproterenol-induced myocardial injury, tanshinone I improves myocardial injury by modulating the Nrf2 signaling pathway.^[40]^ Berberine prevents cardiac remodeling by activating the Nrf2 pathway, reducing doxorubicin-induced oxidative stress and mitochondrial damage.^[50]^ Palmatine improves cardiac remodeling by inhibiting myocardial inflammation and cardiomyocyte apoptosis through activation of the phosphorylated AMP-activated protein kinase/Nrf2 signaling pathway.^[54]^ Daph counters transverse aortic constriction (TAC)-induced cardiomyocyte hypertrophy and fibrosis by enhancing the Nrf2/HO-1 pathway and inhibiting the TGF-β1/Smad2/3 pathway.^[62]^

#### 2.1.2. TGF-β1/Smad signaling pathway

Studies have shown that ginsenoside Rg2 can alleviate myocardial fibrosis by inhibiting TGF-β1/Smad signaling.^[23]^ In a mouse model of pressure overload, cryptotanshinone was shown to improve cardiac remodeling by inhibiting the STAT3 and TGF-β/SMAD3 signaling pathways.^[48]^ Icariin enhances cardiac remodeling by modulating the TGF-β1/Smad signaling pathway.^[55]^ Aloesin reduces isoproterenol-induced cardiomyocyte hypertrophy and fibrosis, protecting against cardiac remodeling by inhibiting the TGF-β/pSmad2/3 pathway.^[61]^

#### 2.1.3. AMPK signaling pathway

Ginsenoside Rb1 was shown to improve myocardial ischemic injury by promoting mitochondrial autophagy through phosphorylation of AMPKα.^[25]^ Isoliquiritigenin inhibits pressure overload-induced cardiomyocyte hypertrophy, also through activating AMPKα.^[36]^ Glycyrrhizin improves pressure overload-induced cardiomyocyte hypertrophy and ameliorates Angiotensin II (Ang II)-induced cardiomyocyte hypertrophy by activating the cAMP/PKA/LKB1/AMPKα2 pathway^[30]^

#### 2.1.4. NF-κB signaling pathway

In a postheart failure MI model, NF-κBp65 signaling suppressed the SERCA2a promoter, resulting in decreased expression levels. However, ginsenoside Rb3 was shown to enhance SERCA2a expression by reducing NF-κBp65 expression.^[27]^ Studies have shown that ginsenoside Re improves cardiac fibrosis by regulating the miR-489/Myd88/NF-κB pathway. In a mouse model of acute myocardial infarction (AMI) and an Ang II-induced cardiac fibroblast model, ginsenoside Re increases the expression of *miR-489*, which inhibits Myd88 and NF-κB expression.^[28]^ In an MI model, tanshinone IIA alleviated myocardial injury by inhibiting the toll-like receptor 4/NF-κBp65 pathway.^[45]^ Oxymatrine was shown to mitigate isoproterenol-induced heart failure by inhibiting the toll-like receptor 4/NF-κB and MAPK pathways.^[60]^ Dihydrotanshinone I reduces doxorubicin-induced cardiac toxicity by modulating the mTOR-TFEB-NF-κB signaling pathway.^[49]^ Furthermore, berberine effectively mitigates myocardial hypertrophy and fibrosis induced by pressure overload by inhibiting the mTOR signaling pathway, significantly improving cardiac remodeling.^[51]^ Recent research identified HHQ16 as an optimized derivative of astragaloside IV. Studies show that HHQ16 binds specifically to lnc4012/lnc9456, leading to their degradation and thereby antagonizing the G3BP2/NF-κB signaling pathway. This finding has been validated in human IncRNAs.^[17]^

#### 2.1.5. Other signaling pathways

Astragaloside IV alleviates myocardial fibrosis and cardiac remodeling induced by MI by inhibiting the ROS/Caspase-1/GSDMD signaling pathway.^[19]^ Additionally, it improves cardiac fibrosis in mice with isoprenaline-induced cardiac fibrosis by enhancing the richness of gut microbiota such as Akkermansia, Defluviitaleaceae_UCG-011, and Rikenella, and boosting amino acid metabolism.^[20]^ The recent research showed that ginsenoside Rg1 significantly improves cardiac remodeling in mice with left anterior descending coronary artery ligation by enhancing silent information regulator 1 (SIRT1)/PTEN-induced putative kinase 1 /Parkin-mediated mitophagy.^[21]^ It also reduces endoplasmic reticulum stress in doxorubicin-induced cardiac toxicity by lowering TIF1 and GRP78 expression and inhibits autophagy by reducing JNK1, P70S6k, and Beclin-1 levels.^[22]^ In a mouse model of aortic arch constriction-induced cardiac hypertrophy, ginsenoside Rg3 improves myocardial hypertrophy by inhibiting the acyltransferase activity of P300, reducing the 2-hydroxyisobutyrylation level of dihydrolipoamide dehydrogenase, and restoring pyruvate dehydrogenase activity.^[24]^ Similarly, ginsenoside Rb1 decreases mitochondrial ROS generation, reduces MI size, and limits myocardial fibrosis, aiding in the myocardial reperfusion injury mitigation.^[26]^ Ginsenoside R1 protects the heart by inhibiting the TAK1-JNK/p38 pathway, thus reducing cardiomyocyte apoptosis and alleviating myocardial ischemia/reperfusion injury.^[29]^ Similarly, in isoproterenol-induced myocardial ischemia in mice, glycyrrhizin reduces intracellular calcium ion concentration by blocking L-type calcium channels, reducing calcium overload, and subsequently alleviating myocardial injury.^[31]^ It also alleviates myocardial ischemia injury by upregulating miR-185-5p and inhibiting CDK6 activity.^[32]^ Glycyrrhetinic acid reduces apoptosis and inflammation induced by endoplasmic reticulum stress in myocardial ischemia by downregulating CHOP, GRP78, and p-PERK expression.^[33]^ It also inhibits the Hippo/Yes-associated protein signaling pathway activation by MI/R, promoting Yes-associated protein nuclear translocation.^[35]^ Tanshinone IIA improves myocardial function by inhibiting the mitochondrial apoptotic signaling pathway, reducing caspase-3, Cyto c, and Apaf-1 expression.^[41]^ It enhances myocardial remodeling by upregulating miR-223-5p, thereby enhancing mesenchymal stem cell-derived exosomes in the heart.^[42]^ It also inhibits doxorubicin-induced cardiomyocyte apoptosis and restores cardiac function by activating the DAXX/MEK/ERK1/2 signaling pathway.^[43]^ Danshenone IIA improves Ang II-induced hypertrophy and myocardial hypertrophy by inhibiting N6-methyladenosine-modified galectin-3.^[44]^ Cryptotanshinone improves doxorubicin-induced cardiac toxicity by reducing oxidative stress and cell apoptosis through the Akt-GSK-3β-mitochondrial permeability transition pore pathway.^[46]^ Cryptotanshinone also enhances MAPK3 expression to inhibit cardiomyocyte apoptosis.^[47]^ Palmatine alleviates doxorubicin-induced myocardial injury and improves cardiac remodeling by inhibiting the STAT3 pathway.^[52,53]^ Large-scale transcriptomic and drug target interaction studies show that luteolin directly interacts with peroxisome proliferator-activated receptor γ, inhibiting its ubiquitination and proteasomal degradation, thereby protecting against cardiac remodeling.^[59]^

In summary, mono-component agents offer diverse mechanisms for treating myocardial remodeling. Despite their lower activity compared with synthetic drugs, combinations of multiple compounds could achieve synergistic effects, enhancing their clinical application value. Future research should explore rational combinations to further benefit heart disease treatment.

### 2.2. Multicomponent agents for improving myocardial remodeling

Multicomponent traditional Chinese medicines effectively treat myocardial remodeling by targeting multiple pathways and mechanisms. These formulations contain diverse active components that interact synergistically, enhancing therapeutic outcomes. Below are some representative examples and their mechanisms of action (as shown in Table 2).

**Table 2:** Multicomponent agents and related pharmacological studies on improving myocardial remodeling.

Drug name	Composition	Animal	Model	Mechanism	References
Optimized New Shengmai Powder	C.P.A., O.R.E.S., R.A., T.S.W., S.M., L.A.W., P.C., C.A.M.	Rats	LADL-induced CH	Myocardial fibrosis↓, Rap1A↓.	^[63]^
Optimized New Shengmai Powder	C.P.A., O.R.E.S., R.A., T.S.W., S.M., L.A.W., P.C., C.A.M.	Rats	LADL-induced CH	MAPK↓.	^[64]^
Sheng Mai Yin	P.G., O.R., S.C.F.	Rats	Dox-induced HF	IL-6↓, TNF-α↓, MMPs ↓, COL-IV↓.	^[65]^
Shenmai Injection	R.G., O.R.	Rats	A high-salt diet-induced HHF	Myocardial fibrosis↓, TGF-β1↓Smad↓.	^[66]^
Baoxin decoction	R.A., F.S.E., S.M., A.S., C.P.A., T.S.W., O.R.C.T.G., S.C.F., P.C.	Rats	DIC	Cardiac function ↑, TGF-β1 ↓.	^[67]^
Yiqi Wenyang (YQWY) decoction	R.A., R.C.R.E.R., A.L.R.P., P.S., L.A.W.,C.R., P.R.A., Z.R.R.	Rats	TAC-induced CH	GATA4↓, MAPKs↑, GATA4↓, ANP↓, BNP↓, and MYH7↓.	^[68]^
Xinfuli Granule	R.A., P.G., S.M., O.R., R.S., L.A.W., P.A., A.N.	Rats	LADL-induced AMI	TGF-β↓, Smads↓.	^[69]^
Qishen granule	R.A., S.M., A.L.R.P., G.G., S.R., H.E.	Mice	DIC	SIRT3↑, Ac-SOD2↑.	^[70]^
Qishen granule	R.A., S.M., A.L.R.P., G.G., S.R., H.E.	Rats	LADL-induced CH	TGF-β↓, Smad3↓, GSK-3β↓.	^[71]^
Qishen granule	R.A., S.M., A.L.R.P., G.G., S.R., H.E.	Rats	LADL-induced CH	(TGF)-β1↓, Smad3 ↓.	^[72]^
Jianxin granule	S.M., R.A., P.S., R.A.S., C.T.G., P.T., L.A.W.,R.G.	Rats	HF	IL-6↓, VEGFA↓, p-AKT1↓.	^[73]^
Kangxian ‐ Yixin Granule	R.G., R.A., S.M., R.A.S., P.C., O.R., C.R.E., L.L., H.L.	Rats	LADL-induced MI	RhoA↓, ROCK1↓.	^[74]^
QiShenYiQi Pill	R.A., S.M., N.R., D.O.	Rats	TAC-induced CH	RP S19↓, TGF-β1↓, FHL2↓.	^[75]^
QiShenYiQi Pill	R.A., S.M., N.R., D.O.	Rats	Cardiac myosin-induced RMF	Beclin-1 ↓, LC3B↓, p62↑, p-PI3K/PI3K↑, p-Akt/Akt↑, p-mTOR/mTOR↑.	^[76]^
QiShenYiQi Pill	R.A., S.M., N.R., D.O.	Rats	TAC-induced CF	Beclin-1 ↓, LC3B↓, p62↑, p-PI3K/PI3K↑, p-Akt/Akt↑, p-mTOR/mTOR↑.	^[77]^
Heart-Protecting Musk Pill	M.K., P.G., S.X., B.R., C.N., V.B., B.L.	Rats	LADL-induced AMI	IL-6↓, TNF-𝛼↓,	^[78]^
Shensong Yangxin capsule	P.G., O.R., S.S., C.O., P.L., E.A.S., N.G., C.C.S., P.P.A., Z.J., S.M., T.E.	Rats	LADL-induced MI	TGF-β1↓, MMP-9↓, TIMP-I↓, Type I and III collagen↓	^[79]^
Qiliqiangxin capsule	R.A., A.L.R.P., P.G., S.M., L.A.W., R.A., C.T., P.C.E., C.T.G., C.P.	Mice	LADL-induced AMI	PPARc↑.	^[80]^
Qiliqiangxin capsule	R.A., A.L.R.P., P.G., S.M., L.A.W., R.A., C.T., P.C.E., C.T.G., C.P.	Rats	TSHRs	α-SMA↓, Collagen I↓, Collagen III↓, TGF-β↓, Bax/ Bcl-2 ↓, PPAR-α↑ PPAR-γ↑, PGC-1α↑.	^[81]^
Taohong siwu decoction	P.P, C.T., R.G.A., P.R.A., L.C.H., A.S.	Mice	LADL-induced MI	Collagen↓, TGFBR1↓, Smad2/3↓.	^[82]^
Tongxinluo	P.G., H.O., P.L., P.C.A., S.N., E.S.A., S.A., L.S.A., L.D.O., F.E., Z.J.B., B.S.	Rats	LADL-induced AMI	EndMT↓, NRG-1↑, ErbB↑, PI3K↑, AKT ↑.	^[83]^
Guanxinshutong capsule	C.A., S.M., S.A., B.L., C.S.B.	Rats	TAC-induced MI	IL-6↓, TNF-α↓, N-cad↑, Cx-43↑, PDGF↓, VEGFA↓, TGF-β1↓, MMP-2↓, MMP-9↓.	^[84]^
Guanxinshutong capsule	C.A., S.M., S.A., B.L., C.S.B.	Rats	TAC-induced CH	TGF-β↓, Smad3↓.	^[85]^
Si-Miao-Yong- decoction	L.J.T., S.N.H., A.S., G.G.	Rats	ISO-induced CH	p38↓, Akt↓.	^[86]^
Si-Miao-Yong-decoction	L.J.T., S.N.H., A.S., G.G.	Mice	TAC-induced CH	TGF-β1↓, Smad↓, TGF-β1↓, TAK1↓, p38↓.	^[87]^
Si-Miao-Yong- decoction	L.J.T., S.N.H., A.S., G.G.	Rats	ISO-induced CF	AMPK↑, Akt↓, mTOR↓, TGF-β↓, SMAD3 ↓.	^[88]^
Qingda granule	G.E.B., U.R., S.B., N.N.	Rats	SHRs	TGF-β1↓, Smad2/3 ↓.	^[89]^
Qingda granule	G.E.B., U.R., S.B., N.N.	Mice	Ang II-induced CH	ROS↓, PI3K↑, AKT↑.	^[90]^
Qingda granule	G.E.B., U.R., S.B., N.N.	Rats	SHRs	NF-Κb↓, DEMs↓.	^[91]^
Guanxin V	S.M., O.R., S.C.F., R.C., C.P.A., P.L.	Mice	LADL-induced AMI	TGF-β1↓.	^[92]^
Guanxin V	S.M., O.R., S.C.F., R.C., C.P.A., P.L.	Mice	LADL-induced VR	TGF-b1-mediated proteasomal degradation of Vimentin↑.	^[93]^
Poge Heart-Saving Decoction	A.L.R.P., Z.O.R., G.G., C.O., P.P.A., C.O.E., M.M., P.G., M.S.	Rats	Dox-induced HF	RAS↓.	^[94]^
Jiajian Yu-Nv-Jian	G.M., A.A.B., S.N.H., R.C., O.R.	Rats	LADL-induced MI	AT1R↓, TNF-α↓, TGF-β1↓, Collagen types I and III↓.	^[95]^
Bu-Yang-HuanWu Decoction	R.A., A.S., P.L., L.C.H., P.P, C.T., L.S.	Rats	TAC-induced CH	Tgf-β↓, Smads↓, MAPKs ↓.	^[96]^
Dan-Qi Soft Capsule	S.M., P.P.V.N.	Rats	LADL-induced MI	TNF-α↓, IL-6↓, TGF-β1↓, TGF-β1↓ Smad 3 ↓.	^[97]^
Dan-Qi Soft Capsule	S.M., P.P.V.N.	Rats	LADL-induced MI	Type I and III collagens↓, α‐SMA↓, TGF‐β1↓, Smad3 phosphorylation↓.	^[98]^
Tong-Guan Capsule	R.A., S.M., H.O., O.R.	Rats	LADL-induced MI	Type I and III collagen↓, α-SMA↓, connexin 43↑.	^[99]^
Tong-Guan Capsule	R.A., S.M., H.O., O.R.	Mice	LADL-induced MI	Sirt1↑.	^[100]^
Ling-Gui-Zhu-Gan Decoction	P.C., C.T.G., R.A.S., G.G.	Rats	LADL-induced MI	Serum ST-2↓, NT-proBNP↓, Wnt3a↓, p-GSK-3β↓, β-catenin↓.	^[101]^
Ling-Gui-Zhu-Gan Decoction	P.C., C.T.G., R.A.S., G.G.	Rats	LADL-induced MI	SIRT1↑, p-AMPK↑,PGC-1α ↑.	^[102]^
Modified Linggui Zhugan Decoction	R.A., P.G., C.T.G., P.C., R.A.S., A.N., S.M., R.A., A.S., L.A.W.	Rats	LADL-induced MI	SIRT3↓.	^[103]^
Lu-Hong Formula	C.N.T., C.T., R.A., S.M., C.C., L.A.W.	Rats	TAC-induced CH	eNOS↑, Col1a1↓, Col3a1↓, TGF-β1↓, caspase-3↓, VEGF↓, VEGFR2 ↓.	^[104]^
Danshen injection	S.M.	Rats	LADL-induced MI	I-NOS↓, MPO ↓, MMP-9 ↓, Bcl-2/Bax ratio↑.	^[105]^
Qi Dan Li Xin pill	R.A., S.M., E.A.F., P.C.	Rats	LADL-induced MI	M-TOR↑, P70S6K ↑.	^[106]^
Yang-Xin-Kang Tablet	P.G., R.A., O.R., S.S., I.P.H.,	Rats	LADL-induced SMI	p-AMPK↓, LC3II/I ↓, Beclin-1↓, p-mTOR↑,p62↑.	^[107]^
Huo-Xue-QianYang Decoction	S.M., H.O., U.R., H.D.R., C.P.B., Z.M.	Rats	High-fat-diet-induced obese SHRs	GRP78↓, ATF6↓, PERK/p-PERK↓, CHOP↓.	^[108]^

↓ indicates a decrease and ↑indicates an increase.

A.A.B. = Anemarrhena asphodeloides Bunge, A.L.R.P. = Aconiti Lateralis Radix Preparata, A.N. = areca-nut, A.S. = Angelica sinensis, AMI = acute myocardial ischemia, B.L. = Borneol, B.R. = Bezoar, B.S. = Borneolum syntheticum, C.A. = Choerospondias axillaris, C.A.M. = Citrus aurantium, C.C. = Cinnamomum cassia, C.C.S. = Coptis chinensis, C.N. = Cinnamon, C.N.T. = Cervus nippon Temminc, C.O. = Cornus officinalis, C.O.E. = Concha Ostreae, C.P. = Cortex Periplocae, C.P.A. = Codonopsis pilosula, C.P.B. = Crataegus pinnatifida Bunge, C.R. = Curcumae Rhizome, C.R.E. = Cimicifugae Rhizome, C.S.B. = concretio silicea bambusae, C.T. = Carthamus tinctorius, C.T.G. = Cassia twig, CF = cardiac fibrosis, CH = cardiac hypertrophy, DIC = doxorubicin-induced cardiomyopathy, D.O. = Dalbergia odorifera, Dox = doxorubicin, E.A.F. = Epimedium acuminatum Franch, E.A.S. = Eupolyphaga sinensis, E.S. = Eleutherococcus senticosus, E.S.A. = Eupolyphaga Steleophaga, F.E. = Frankincense, F.S.E. = Forsythia suspense, G.E.B. = Gastrodia elata Blume, G.G. = Glycyrrhiza glabra, G.M. = Gypsum, H.D.R. = Haliotis diversicolor Reeve, H.E. = Honeysuckle, H.L. = Herba Leonuri, H.O. = Hirudo, HHF = hypertensive heart failure, IL = interleukin, I.P.H. = Ilex pubescens Hook, ISO = isoprenaline, L.A.W. = Lepidium apetalum Willd, L.C.H. = Ligusticum chuanxiong Hort, L.D.O. = Lignum dalbergiae odoriferae, L.J.T. = Lonicera japonica Thunb, L.L. = Lycopus lucidus, L.S.A. = Lignum santali albi, LADL = left anterior descending coronary artery ligation, MI = myocardial infarction, M.K. = Musk, M.M. = Magnetitum, MMP-2 = matrix metallopeptidase 2, MMP-9 = matrix metallopeptidase 9, MPO = myeloperoxidase, M.S. = Moschus, N.G. = Nardostachys grandiflora, N.N. = Nelumro nucifera, N.R. = notoginseng radix, O.R. = Ophiopogonis Radix, O.R.E.S. = Ophiopogonis Radix Eleutherococcus senticosus, P.A. = papaya, p-AMPK = phosphorylated AMP-activated protein kinase, P.C. = Poria cocos, P.C.A. = Periostracum cicadae, P.C.E. = Pericarpium Citri Reticulatae, PDGF = platelet-derived growth factor, P.G. = Panax ginseng, P.L. = Paeonia lactiflora, P.P = Prunus persica, P.P.A. = Polypodium pseudo-amoenum, P.P.V.N. = Panax pseudoginseng var. Notoginseng, P.R.A. = Paeoniae Radix Alba, P.S. = Polyporus, P.T. = Pollen Typhae, PPAR = peroxisome proliferator-activated receptor, R.A. = Radix Astragali, R.A. = Rhizoma Alismatis, R.A.S. = Rhizoma Atractylodis, R.C. = Rehmannia chingii, R.C.R.E.R. = Rhodiolae Crenulatae Radix et Rhizoma, R.G. = Red ginseng, R.G.A. = Rehmannia glutinosa, R.S. = Rhizoma Sparganii, RAS = renin-angiotensin system, RMF = reparative myocardial fibrosis, S.A. = Scolopendra, S.A. = Syzygium aromaticum, S.B. = Scutellaria baicalensis, S.C.F. = Schisandrae Chinensis Fructus, SIRT1 = silent information regulator 1, S.M. = Salvia miltiorrhiza, S.N. = scorpion, S.N.H. = Scrophularia ningpoensis Hemsl, S.R. = Scrophulariae Radix, S.S. = Schisandra sphenanthera, S.X. = storax, T.E. = Taxillus estipitatus, TGF-β1 = transforming growth factor beta 1, T.S.W. = Trionyx sinensis Wiegmann, TAC = transverse aortic constriction, TSHRs = thirteen-week-old spontaneously hypertensive, U.R. = Uncaria rhynchophylla, V.B. = Venenum Bufonis, VEGFA = vascular endothelial growth factor A, VR = ventricular remodeling, Z.J. = Ziziphus jujuba, Z.J.B. = Zizyphus jujube benevolence, Z.M. = Zea mays, Z.O.R. = Zingiber officinale Roscoe, Z.R.R. = Zingiberis Rhizome Recens.

#### 2.2.1. The TGF-β1/Smad signaling pathway

Shenmai injection inhibits myocardial fibrosis by regulating the TGF-β1/Smad signaling pathway, effectively improving hypertensive heart failure.^[66]^ Baoxin decoction effectively alleviates doxorubicin-induced dilated cardiomyopathy in rats by inhibiting the TGF-β1 signaling pathway.^[67]^ Xinfu Liqi granules improve ventricular remodeling and inhibit myocardial fibrosis in AMI rats by regulating the TGF-β/Smads signaling pathway.^[69]^ In addition, Qishen granules prevent myocardial fibrosis by suppressing the TGF-β/Smad3 pathway and GSK-3β phosphorylation, also targeting fibrosis activated by M1 macrophages.^[71,72]^ Qishen Yiqi dropping pills effectively alleviate myocardial fibrosis by regulating the FHL2 and RP S19-TGF-β1 signaling pathways.^[75]^ Shensong Yangxin capsules improve left atrial fibrosis by inhibiting the expression of TGF-β1, matrix metallopeptidase 9 (MMP-9), TIMP-I, and collagen types I and III, and preventing cardiomyocyte transformation into myofibroblasts.^[79]^ Furthermore, Qili Qiangxin capsules reduce cardiac fibrosis by downregulating α-SMA, collagen I, collagen III, and TGF-β 1, and improving the Bax/Bcl-2 ratio.^[81]^ Tao Hong Si Wu Decoction alleviates myocardial fibrosis by inhibiting the TGF-β1 pathway.^[82]^ Guanxin Shutong capsules improve cardiac function postinjury by reducing ventricular remodeling and inhibiting platelet-derived growth factor, TGF-β1, vascular endothelial growth factor A, matrix metallopeptidase 2, and MMP-9 while increasing N-cadherin and Cx-43.^[84]^ In addition, they also inhibit myocardial fibrosis by suppressing the TGF-β/Smad3 pathway.^[85]^ Furthermore, Simiao Yong’an Decoction improves cardiac fibrosis by inhibiting the TGF-β1/Smad and TGF-β1/TAK1/p38 signaling pathways,^[87]^ and alleviates isoproterenol-induced fibrosis by regulating AMPK to induce Akt/mTOR and TGF-β/Smad3 pathways.^[88]^ Qingda granules significantly alleviate myocardial fibrosis by inhibiting the TGF-β1/Smad2/3 signaling pathway.^[89]^ Guanxin V effectively alleviates oxidative stress injury, apoptosis, and fibrosis in AMI by inhibiting the TGF-β1 pathway^[92]^ and promoting Vimentin degradation via the ubiquitin-proteasome pathway.^[93]^ Jiajian Yunvju decoction improves cardiac remodeling by inhibiting RAS, and downregulating AT1R, TNF-α, and TGF-β1.^[95]^ Buyang Huanwu Decoction protects against pressure overload-induced cardiac remodeling by inhibiting TGF-β/Smads and MAPKs signaling pathways.^[96]^ Danqi Soft Capsules reduce AF and cardiac fibroblast proliferation in rats by inhibiting the TGF-β1/Smad3 pathway, improving interstitial fibrosis and myocardial hypertrophy in MI.^[97,98]^ The Luhong Formula ameliorates left ventricular remodeling in pressure overload heart failure models through the upregulation of eNOS and the downregulation of Col1a1, Col3a1, TGF-β1, caspase-3, VEGF, and VEGFR.^[104]^

#### 2.2.2. Akt-mTOR signaling pathway

In an effort to study autoimmune myocarditis, a rat model exhibiting myocardial fibrosis was created by injecting myosin into the myocardium. Qishen Yiqi pills were shown to alleviate the resulting myocardial fibrosis by altering the activation of the p-PI3K/Akt-mTOR signaling pathway, preventing excessive autophagy.^[76]^ Similarly, in a rat myocardial fibrosis model induced by restricting blood flow through the aorta, Qishen Yiqi pills could also alleviate myocardial fibrosis through a similar mechanism.^[77]^ Qidan Lixin pills improved cardiac remodeling, by regulating autophagy mediated by mTOR/p70S6k, and inhibited apoptosis.^[106]^ Yangxin Kang tablets were shown to reduce cardiac fibrosis and prevent cardiomyocyte apoptosis by inhibiting AMPK/mTOR signaling through suppressing autophagy.^[107]^ Jianxin Granules improved cardiac function and reduced myocardial fibrosis by regulating the expression of IL-6, vascular endothelial growth factor A, and p-AKT1.^[73]^ Research has demonstrated that Tongxinluo enhances myocardial fibrosis in a mouse model of myocardial ischemia-reperfusion by activating the PI3K/AKT pathway, thus inhibiting endothelial-to-mesenchymal transition.^[83]^ Most of the miRNAs activate the PI3K/AKT pathway, thus inhibiting endothelial-to mesenchymal transition.^[109]^ Simiao Yong’an decoction improves isoproterenol-induced myocardial hypertrophy and fibrosis by inhibiting the phosphorylation of p38 and Akt.^[86]^

#### 2.2.3. SIRTs-related signaling pathway

The herbal preparation, Qi Shen Granule, was shown to prevent oxidative damage and support mitochondrial function through restoring protein acetylation levels and activating the SIRT3/Ac-SOD2 pathway.^[70]^ Additionally, Tong Guan Capsule could prevent cardiac remodeling following MI in mice by inhibiting cardiomyocyte inflammation and apoptosis, as well as by activating Sirt1 to enhance autophagy.^[100]^ The traditional Chinese herbal decoction Ling Gui Zhu Gan Tang has been shown to effectively reduce cardiomyocyte hypertrophy and fibrosis by inhibiting the Wnt/β-catenin signaling pathway, thus protecting against cardiac remodeling.^[101]^ In addition, Ling Gui Zhu Gan Tang was also shown to enhance the antioxidant capacity of cardiomyocytes and protect mitochondrial function by activating the SIRT1/AMPK/PGC-1α signaling pathway.^[102]^ Similarly, a modified version of Ling Gui Zhu Gan Tang was shown to improve ventricular remodeling following MI in rats by preventing mitochondrial damage and associated apoptosis. This protective effect was shown to function through SIRT3.^[103]^

#### 2.2.4. TNF and NF-κB signaling pathway

The herbal formulation Sheng Mai Yin has been shown to attenuate adriamycin-induced myocardial injury by inhibiting the expression of IL-6 and TNF-α, as well as suppressing the overexpression of MMPs and COL-IV. These alterations to gene expression improve cardiac remodeling.^[65]^ The traditional Chinese medicine Shexiang Baoxin Wan has been shown to improve cardiac function and attenuate ventricular remodeling by inhibiting the expression of IL-6 and TNF-α.^[78]^ Leonurus heterophyllus reduces cardiomyocyte hypertrophy and fibrosis by inhibiting the MAPK and NF-κB pathways and improves fibrosis by regulating the p53/miR-29a-3p pathway to reduce collagen synthesis.^[56–58]^ Additionally, Qing Da Granules significantly attenuated cardiac remodeling and inflammation in spontaneously hypertensive rats by inhibiting the NF-κB pathway.^[91]^

#### 2.2.5. Other signaling pathways

Recent research indicates that the optimized formulation of the traditional Chinese herbal medicine Xin Sheng Mai San can mitigate myocardial fibrosis in rat models of heart failure by regulating the cAMP/Rap1 pathway. This treatment strategy results in the improvement of cardiac remodeling in rat models of heart failure.^[63]^ The optimized Xin Sheng Mai San formulation has also demonstrated potential in alleviating myocardial injury in heart failure rat models by inhibiting the MAPK signaling pathway.^[64]^ The traditional Chinese herbal formula Yi Qi Wen Yang Tang was shown to inhibit hypertrophic remodeling due to TAC through suppressing GATA4 and MAPK phosphorylation. This inhibition subsequently resulted in decreased pathogenic remodeling.^[68]^ The herbal formulation Kang Xian Yi Xin Granules was shown to effectively alleviate myocardial fibrosis in rat models of dilated cardiomyopathy by inhibiting the RhoA/ROCK1 signaling pathway.^[74]^ Qi Li Qiangxin capsules were shown to enhance peroxisome proliferator-activated receptor γ levels, effectively reducing cardiac remodeling after AMI.^[80]^ The herbal formulation, Qing Da Granules, ameliorated cardiac hypertrophy induced by Angiotensin II and prevented apoptosis, by stimulating PI3K/AKT pathway activation and preventing the accumulation of ROS.^[90]^ Po Ge Jiu Xin Tang, a traditional Chinese herbal formula, has been demonstrated to enhance cardiac function and reverse ventricular remodeling in rats with adriamycin-induced heart failure by blocking the RAS pathway.^[94]^ Tong Guan Capsule has been found to prevent remodeling in the MI border zone by inhibiting fibroblast differentiation. This leads to reduced interstitial fibrosis and decreased susceptibility to rapid arrhythmias in the affected area.^[99]^ Salvia miltiorrhiza injections had anti-inflammatory activity through reducing the expression of inducible nitric oxide synthase and myeloperoxidase, resulting in attenuated myocardial fibrosis by decreasing the expression of MMP-9. This treatment enhanced apoptosis by increasing the Bcl-2/Bax ratio.^[105]^ The traditional Chinese formula Huo Xue Qian Yang Fang has been found to significantly improve cardiac remodeling in obese spontaneously hypertensive rats by inhibiting the ATF6-CHOP endoplasmic reticulum stress signaling pathway.^[108]^

In summary, multicomponent traditional Chinese medicine formulas provide diverse options for treating myocardial remodeling through various mechanisms and pathways. Clinical studies suggest that the synergistic effects of the compatible monomer components in these polyherbal preparations enhance their therapeutic potential. This underscores the value of these compound medications for preventing pathological myocardial remodeling and opens new avenues for pharmaceutical research and development. Despite the complex mechanisms of action, advancements in science and technology and a deeper understanding of the pharmacology of these herbs will likely increase the significance of these medications in heart disease treatment, offering greater benefits to patients.

## 3. Discussion

This review summarizes recent advances in the treatment of pathological remodeling of the myocardium, describing both the application and mechanisms of action for both single-component and multicomponent agents. Single-component agents, including astragaloside IV and ginsenoside Rg1, present a variety of treatment approaches to combat myocardial remodeling through diverse mechanisms and pathways. However, natural products often have lower potency compared to synthetic chemicals, which diminishes their likelihood of direct clinical application. The therapeutic limitations of single-target agents—including suboptimal bioavailability due to rapid metabolism and membrane impermeability, as well as restricted efficacy stemming from pathway redundancy—highlight the necessity of embracing network pharmacology principles in myocardial remodeling treatment.

**Figure 1.**
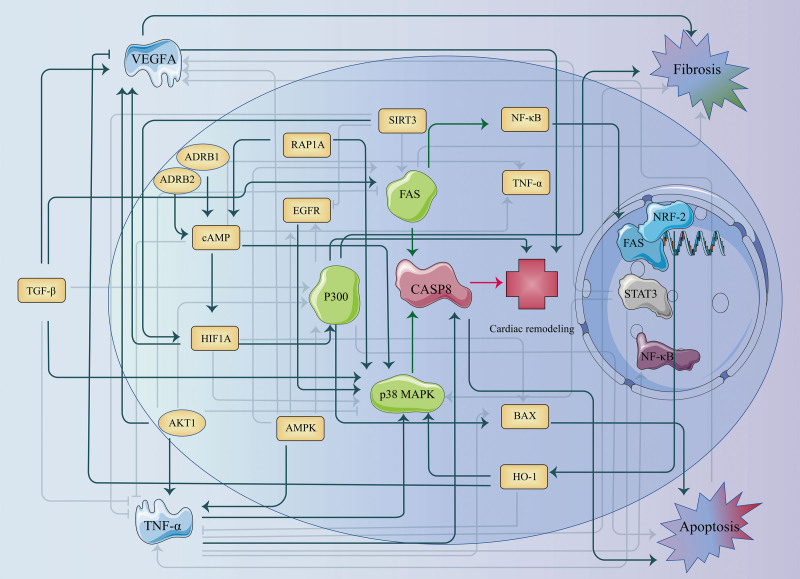
Main pathways and core signal transduction molecules in cardiac remodeling. ADRB = beta-adrenergic receptor, AMPK = AMP-activated protein kinase, BAX = BCL-2 associated X protein, cAMP = cyclic adenosine monophosphate, CASP8 = caspase 8, EGFR = epidermal growth factor receptor, HIF1A = hypoxia-inducible factor 1-alpha, HO-1 = heme oxygenase-1, NF = nuclear factor, RAP1A = a small GTPase involved in cell adhesion, cell migration, and gene expression regulation, SIRT3 = sirtuin 3, TGF = transforming growth factor, TNF = tumor necrosis factor, VEGFA = vascular endothelial growth factor A.

Recent studies, including the work by Rai and colleagues,^[110]^ suggest that multicomponent herbal formulations could achieve greater efficacy in the treatment of myocardial remodeling by leveraging the synergistic interactions between various constituents. These interactions may enhance the therapeutic outcome by targeting multiple pathways simultaneously, thereby providing a more comprehensive approach to cardiovascular disease management. Emerging evidence suggests that multicomponent herbal formulations (e.g., Xin Sheng Mai San, Sheng Mai Yin) exert their superior clinical effects not through random compound combinations, but via orchestrated modulation of evolutionarily conserved critical signaling hubs. These hubs extend beyond VEGF and TNF-α to include master regulators like TGF-β, NF-κB, and miRNA networks, which form an interconnected signaling lattice governing fibrotic, inflammatory, and metabolic reprogramming (Fig. 1). These hubs, exemplified by VEGF, TNF-a, and Caspase-3, function as convergent nodes that gatekeep crosstalk between fibrotic, inflammatory, and metabolic pathways. TGF-β/Smad3 signaling emerges as a central conductor of fibrotic remodeling. TGF-β1 activates both canonical (Smad2/3) and noncanonical pathways (MAPK, PI3K/Akt), driving fibroblast-to-myofibroblast transdifferentiation and excessive collagen deposition.^[111]^ This explains why multitarget agents like QLQXC, which modulate TGF-β crosstalk with TNF-α and NF-Κb,^[112]^ show superior clinical efficacy compared with single-pathway inhibitors. NF-κB operates as the inflammatory lynchpin connecting cytokine storms to fibrotic progression. TNF-α-induced NF-κB activation not only amplifies IL-6/IL-1β production,^[113]^ but also directly phosphorylates Smad3 to synergize with TGF-β-driven fibrosis.^[114]^ This creates a feedforward loop where NF-κB + macrophages transition infarct zones from inflammatory to fibrotic phases. Notably, the RelB subunit of NF-κB shows particular promise as a therapeutic target, as its inhibition reduces fibrotic burden without compromising acute inflammation resolution.^[115]^ miRNAs have emerged as fine-tuners of remodeling pathways at multiple hierarchical levels. For instance, miR-21 sustains fibroblast activation via PTEN/Akt signaling, with preclinical antagomirs showing 40% fibrosis reduction^[116]^; miR-34a promotes cardiomyocyte senescence by suppressing SIRT1, correlating with diastolic stiffness in human heart failure with preserved ejection fraction^[117]^; miR-199a-5p elevation post-MI predicts adverse remodeling through Bcl-2-mediated apoptosis resistance.^[118]^ These miRNA networks interface with discussed hubs-TGF-β/Smad pathway upregulated by miR-21, while NF-κB transcriptionally activates miR-34a clusters.^[119]^

Modern pharmacology suggests that targeting these key nodes may lead to more efficient improvements in cardiac remodeling. Notably, critical signaling hubs exhibit hierarchical regulatory patterns. Taking TNF-α as an example, this pleiotropic cytokine (a) activates NF-KB via TNFR1 to induce inflammatory responses,^[113]^ (b) modulates apoptosis through FADD/caspase-8 axis,^[120]^ and (c) cross-talks with TGF-β1/Smad3 pathway to promote extracellular matrix deposition.^[111]^ Such multidimensional interactions explain why TNF-α inhibitors show superior efficacy in diastolic dysfunction compared to single-pathway modulators.

The first-line drug sacubitril/valsartan demonstrates this principle by enhancing natriuretic peptides (cGMP/PKG-mediated Smad3 inhibition) while suppressing angiotensin II (NF-κB activation), it concurrently targets fibrotic and inflammatory axes.^[121]^ Similarly, QLQXC’s clinical success in the QUEST trial^[112]^ likely stems from its multihub regulation, reducing miR-34a overexpression while normalizing TGF-β/NF-κB crosstalk in heart failure with reduced ejection fraction patients. Despite widespread reports of Caspase inhibitors having antiapoptotic and antiheart failure effects, no mature clinical-stage drugs or drugs in development possess these specific attributes. Based on the aforementioned findings, traditional medicines that modulate miRNAs, or herbal formulas containing these herbs, may exhibit broader clinical applicability. Furthermore, natural products capable of regulating the aforementioned signaling networks (e.g., TGF-β/Smad3, NF-κB, and miRNA cascades) still hold significant potential for drug development by targeting key signaling hubs involved in fibrotic and inflammatory pathways.

A comprehensive review of the literature reveals that cardiac remodeling is a highly complex process influenced by a multitude of factors. It involves the intricate interaction and regulation of numerous molecules and signaling networks rather than the alteration of a single pathway or target. Therefore, therapeutic strategies for cardiac remodeling should not focus solely on single-target modulation. The QUEST study, led by Professor Li Xinli, provides robust evidence for the efficacy of QLQXC in the treatment of heart failure with reduced ejection fraction. This multicenter trial, encompassing 3110 patients from 133 hospitals across mainland China and Hong Kong, spanned an observation period of 12 to 36 months.^[112]^ The study’s findings are significant: QLQXC, when administered in conjunction with standard heart failure therapy, demonstrated a 24% reduction in the risk of hospital readmission due to heart failure exacerbation and a 17% reduction in the risk of cardiovascular mortality, compared with placebo plus standard therapy.

## 4. Future perspectives

There are several inherent challenges with the development multicomponent drug therapies for myocardial remodeling. First, identifying biologically active compounds and defining their mechanism of action is a necessary first step. Second, the precise determination of component proportions is critical for maximizing the therapeutic efficacy of drug combinations. Additionally, off-target effects become a more pronounced concern with multicomponent agents, as the combination of compounds may interact in unforeseen ways, leading to adverse reactions and reducing the therapeutic index. The challenge of optimizing the ratio and dosage of each component further complicates the development and standardization of these agents. To address this challenge, we propose the integration of several advanced strategies and technologies. Systems pharmacology offers a holistic approach by modeling the complex interactions between multiple compounds, allowing for the prediction of optimal dosing ratios based on pharmacokinetic and pharmacodynamic data. High-throughput screening techniques enable the rapid evaluation of a vast array of component ratios, identifying those that elicit the most favorable biological responses. Additionally, AI-driven modeling employs machine-learning algorithms to analyze large datasets and predict the optimal combination of components, thereby streamlining the process of drug formulation and enhancing the potential for therapeutic success.

The advent of advanced omics technologies, including genomics, proteomics, and metabolomics, has significantly enhanced our ability to pinpoint specific targets and pathways involved in myocardial remodeling. For instance, the combination of single-cell sequencing with proteomic analyses allows for a more precise and comprehensive identification of therapeutic targets.^[122]^ To address the intricate nature of myocardial remodeling and advance the development of multicomponent therapies, a comprehensive and integrated approach is essential. The convergence of bioinformatics, computational biology, and medicinal chemistry offers a powerful framework for tackling the complexity of this disease process.

The transition from preclinical to clinical settings for multicomponent agents is fraught with hurdles. Regulatory considerations demand rigorous toxicological and pharmacokinetic studies to ensure safety and efficacy. Manufacturing processes must be developed to maintain consistency in composition and quality, which is particularly challenging for complex mixtures. More critically, it is essential to thoroughly understand the potential toxicity and chemical interactions between multiple components, as these factors could significantly impact the drug’s efficacy.^[123,124]^ Additionally, the standardization of natural products is critical to ensure batch-to-batch uniformity and to meet regulatory requirements. Safety considerations include the potential for drug interactions and the need for comprehensive monitoring of adverse effects. Addressing these challenges is essential for the successful translation of multicomponent agents from the laboratory to the clinic, thereby bridging the gap between traditional practices and modern medicine.

Future research should determine how to combine Chinese medicine with modern medical approaches in order to improve clinical efficacy for treating myocardial remodeling. Additionally, in-depth studies on the mechanism of action of traditional Chinese medicine formulations will be essential for understanding their therapeutic utility and potential side effects. Through these efforts, we can optimize the use of traditional Chinese medicine formulations and develop new compound drugs, providing patients with safer and more effective treatment options. With continued technological progress, traditional Chinese medicine is poised to play a more significant role in the treatment of myocardial remodeling, ultimately providing greater benefits to patients.

## Author contributions

**Writing—original draft:** Jimin Liu, Taiyi Wang.

**Investigation:** Xiaqing Tian.

**Writing—review & editing:** Meng Zhang, Yongjun Chen, Taiyi Wang.

**Data curation:** Jiaxuan Li.
